# Effects of pre-conditioning on behavior and physiology of horses during a standardised learning task

**DOI:** 10.1371/journal.pone.0174313

**Published:** 2017-03-30

**Authors:** Kate Fenner, Holly Webb, Melissa J. Starling, Rafael Freire, Petra Buckley, Paul D. McGreevy

**Affiliations:** 1 School of Animal and Veterinary Sciences, Charles Sturt University, Wagga Wagga, NSW, Australia; 2 Faculty of Veterinary Science, University of Sydney, NSW, Australia; Tokai University, JAPAN

## Abstract

Rein tension is used to apply pressure to control both ridden and unridden horses. The pressure is delivered by equipment such as the bit, which may restrict voluntary movement and cause changes in behavior and physiology. Managing the effects of such pressure on arousal level and behavioral indicators will optimise horse learning outcomes. This study examined the effect of training horses to turn away from bit pressure on cardiac outcomes and behavior (including responsiveness) over the course of eight trials in a standardised learning task. The experimental procedure consisted of a resting phase, treatment/control phase, standardised learning trials requiring the horses (n = 68) to step backwards in response to bit pressure and a recovery phase. As expected, heart rate increased (*P* = 0.028) when the handler applied rein tension during the treatment phase. The amount of rein tension required to elicit a response during treatment was higher on the left than the right rein (*P* = 0.009). Total rein tension required for trials reduced (*P* < 0.001) as they progressed, as did time taken (*P* < 0.001) and steps taken (*P* < 0.001). The incidence of head tossing decreased (*P* = 0.015) with the progression of the trials and was higher (*P* = 0.018) for the control horses than the treated horses. These results suggest that preparing the horses for the lesson and slightly raising their arousal levels, improved learning outcomes.

## Introduction

Training can be stressful for horses but little is known about how best to prepare them for effective learning. Some riders currently prepare their horses to be ridden by chasing them around the round pen [1] and lunging [2]. Unfortunately, these exercises are designed to tire, rather than to mentally engage, the horse and can possibly therefore compromise learning from the outset of the lesson. Human studies reveal that cognitive impairment results from both anxiety-induced increases in arousal level and exercise-induced fatigue [3]. While exercise-induced fatigue has been reported to produce both positive and detrimental cognitive effects [4], it is known that chasing the horse should always be avoided [5]. Inducing fear prior to learning is not commensurate with good training but is encouraged by some of the world’s most popular trainers [2].

Learning theory describes how animals absorb, process and retain information through stimulus-response-reinforcement chains [6, 7], together with the emotional and environmental factors that influence this [8]. Many horse trainers and equestrian coaches are unaware of how horses learn or of the mechanisms that underpin positive and negative reinforcement and punishment [9]. This knowledge gap compromises coaches’ ability to study the emergent equitation science literature and to convey information about learning theory and training to students and horse owners [9].

Riders and handlers need to understand the relationship between distress and cognition, to recognise events that horses may find stressful and to identify behavioral responses that suggest that a horse is overly emotional. These clues tell trainers when to reduce stressors in training. Emotional reactivity refers to physiological, chemical and behavioral changes resulting from so-called emotional stimulation [10]. Such stimulation can arise from a variety of novel or otherwise potentially frightening stimuli in the horse’s environment. We know that emotionally reactive horses can be difficult to handle [11] and horse-human relationships improve when the horses’ emotional reactivity is minimized [12]. Repeated exposure to increases in emotional level impairs cognitive function [13]. However, any such impairment is attenuated when the stressor is predictable [14]. The opposite of a highly emotionally reactive horse may be one that is either inherently relaxed, has been systemically desensitized to pressure, or may even be in a state of learned helplessness as a result [15]. Learned helplessness refers to a state where the horse has repeatedly been unable to escape aversive stimuli, such as bit or leg pressure, and eventually simply stops trialling any behaviors in an attempt to do so [15].

Any apparatus that applies pressure or constrains a horse’s movement has the potential to compromise welfare [16]. In equitation, increases in rein tension primarily provide directional and deceleration cues from the handler or rider to the horse and, secondly, modify the head and neck position [17]. Reins tension converts to pressure on various parts of the horses’ mouth and face and the timely release of this pressure communicates to the horse that it has made the correct response. This process is known as negative reinforcement, which is the removal of aversive stimuli to reward a desired response [18]. So, it is recommended that rein tension be applied minimally and consistently and as appropriate to the task. For effective and humane negative reinforcement, pressure-release relies on good timing and consistency [19]. Inconsistency and delays in timing may inadvertently punish the horse, untrain established responses and impede the acquisition of desirable ones [20].

For most horses, accommodating the bit is uneventful and they become habituated to having the bit in their mouth [21]. While habituation to the presence of the bit is desirable, habituation to additional bit pressure from rein use compromises effective training because it means that horses no longer respond to light pressure. This can lead to the need for excessive rein use and severe bits [19] that can threaten horse welfare. Mean rein tension ranges have been reported between 3 to 20 N by Heleski, McGreevy, Kaiser, Lavagnino, Tans, Bello and Clayton [17], but higher ranges of 40 to 75 N have also been reported [22, 23]. To avoid habituation to bit pressure and assure animal welfare, rein tensions used in training and competition should be kept to a minimum.

Previous rein tension studies have described the role rein cues play in training horses to slow down [24] and avoid bit pressure [18] but none have reported the physiological and behavioral responses that accompany increased rein tension during a standardised learning task. Physiological parameters, such as heart rate, in combination with behavioral scoring may help to reveal the complexity of equine responses to bit pressure. Such responses may have important implications for welfare during training and competition [25]. Equally, some physiological and behavioral responses are of importance to competitors since they may compromise performances.

The aim of this study was to determine the effect of a pre-conditioning exercise, namely giving to bit pressure, on arousal level (measured by heart rate and behavior) and learning outcomes (measured by rein tension, time, step number and behavior) of horses. The pre-conditioning treatment was designed to *engage* the horse with the lesson. The purpose of the treatment was to achieve a moderately elevated, stable heart rate that was maintained throughout the pre-conditioning and subsequent trials, followed by a return to resting levels at the conclusion of the trial phase. It was hypothesized that the pre-conditioning would prepare the horses to respond appropriately to bit pressure and improve their performance in the trials that immediately followed.

## Materials and methods

The protocol and conduct of this study were approved by the Charles Sturt University Animal Care and Ethics Committee, New South Wales, Australia (ACEC protocol number 14/030).

### Selection of horses

Eligible horses were selected from a population of riding horses. Most of the selected horses had been previously been taught to back-up from pressure on the nose with a head collar, two reins together from the ground or saddle, voice cues as well as other cues, such as tapping on the forelegs or chest. However all of them were naive to backing from rein tension from a single rein in the absence of all other signals. Horses were randomly assigned to either the treatment or control group, using an Excel generated randomization spreadsheet. They were recreational, pleasure riding or companion horses, and were not engaged in high-level competition. Horses that were active in adult riding club, pony club, trail riding, and riding school activities and that were habituated to wearing a girth and a bridle with a simple snaffle bit were eligible for inclusion.

Sixty-eight horses of various breeds were selected for the study. They comprised 35 geldings, 4 stallions and 29 mares. Horses had mixed training histories, ranging from a basic start to many years of consistent training (mean duration of training 3.1 ± 2.3 years). They were between 94.5 cm (9.3 hh) and 174.8 cm (17.2 hh) in height with a mean height of 149.3 ± 15.24 cm (14.3 ± 1.2 hh), and were aged from 2 to 25 years (mean age 9.8 ± 6.1 years). Sixty-two of the horses were housed in paddocks full-time, while six occasionally spent either the day or the night in a stable.

### Data collection

Data collection took place in an arena or property that was familiar to each horse. Fenced arenas were used where available and a safe, flat, fenced area, familiar to the horses, was chosen if no arena was available.

**Fig 1:**
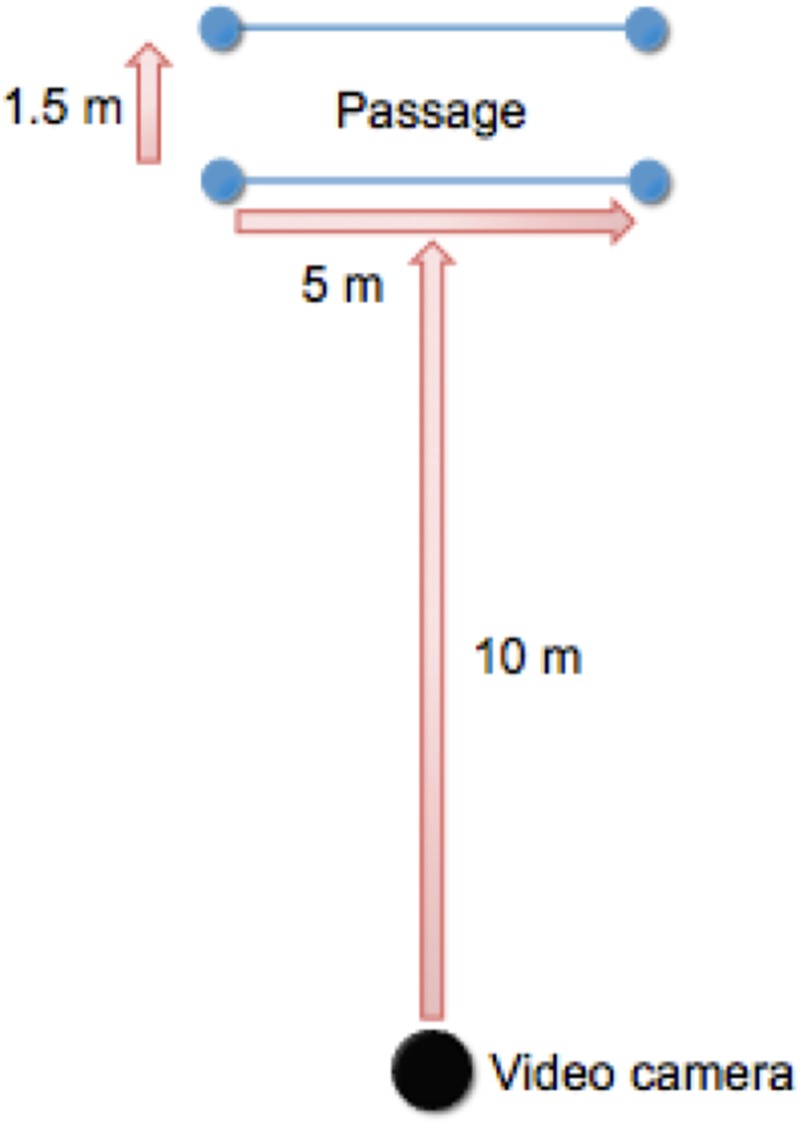
Experimental area. The passage was 5 m x 1.5 m. The camera was 10 m from the passage and turned as the horse moved between phases.

Immediately prior to data collection, each horse was fitted with a Centaur Trainology Rein Tension Device (Centaur Consulting, Vijverhof 27, 3734 DB Den Dolder, The Netherlands) attached to a full-cheek snaffle bit, held in place by an open bridle with no throatlatch, brow band or noseband. A webbing head collar was fitted underneath the bridle and leather reins were connected to the bit via the rein tension device. A surcingle was fastened on each horse and a Polar heart rate monitor (RS800CX) was attached with electrode pads placed under the surcingle with one behind the left elbow and the other at the withers. Video recordings were taken for each horse for the entire duration of the procedure. A Sony HDR-PJ790 camera (Sony Corporation of America, Sony Drive, Park Ridge, NJ 07656, USA), placed 10 m from the experimental area, was panned horizontally as the horse moved between experimental phases (see Fig 1). Baseline and recovery phases took place in the same area, approximately 10m from the test corridor. Each of the four experimental phases was recorded, resulting in a total of approximately 30 minutes of footage per horse.

#### Behavior observations

A behavioral observation sheet was developed for this study (see Table 1). Behavior was counted and coded using the Observer XT (Noldus Information Technoloty bv, Wageningen, The Netherlands, v11.5, 2013). A possible limitation of this study is that the person scoring the observer was not blinded to the treatments. The videos were divided into resting, treatment/control, trials and recovery phases and all of the trial phases were scored initially, followed by the recovery, resting and finally the treatment phases. In addition, a second person also scored a random sample of 20 videos that matched the initial findings. It was concluded that this resulted in a very low risk of bias. An improvement in future studies could be to have the all scoring completed by a person blinded to treatment.

**Table 1:** Behavioral observation sheet developed for this study.

**Behavior**	**Definition**
Head toss	Quick upward vertical movement of the head.
Handler contact	Touching the handler with the muzzle.
Paw	Hoof being lifted off the ground and extended forward before the toe is quickly dragged backward against the ground.
Yawn	Deep inhalation with the mouth wide open and jaws either opened or moved from side to side.
Chew on bit	Licking of the lips and opening the mouth so that a gap is apparent between the mandible and maxilla.

#### Heart rate

Horse heart rate was recorded at one-second intervals for the entire experimental period, starting with the resting phase and finishing when the recovery phase was completed. The data were stored in the Polar unit and later downloaded to the Polar Equine Pro Trainer 5 software for analysis.

#### Rein tension

Rein tension data, expressed in Newtons (N), were sampled at a rate of 100 Hz with a resolution of 21.6 +/- 1.8 Newton/Bits. This resulted in a large number of readings per horse from which one reading per second was taken for the purposes of this analysis. This was done by extracting each of the one hundredth readings, giving one reading per second. The rein tension monitors were calibrated at the start of each testing day. The data were live-streamed to a Windows based Hewlett Packard computer (HP Inc., 1501 Page Mill Road, Palo Alto, CA 94304, USA) within the testing area.

### Experimental procedure

#### Resting phase

The horse was led from the stable block to the experimental area, using a webbing head collar and lead rope. The horse was bridled with rein tension monitors linking the reins to the bit and the reins were placed over the horse’s neck. The video recorder and heart rate monitor were activated and the resting phase began. The handler loosely held the horse by a lead rope for 5 minutes.

#### Treatment and control phase

The 31 horses allocated to the treatment group were pre-conditioned with a negatively reinforced exercise known as ‘give-to-the-bit’ [26], a simple pressure-release activity that was repeated for 8 minutes. Pilot trials revealed that 8 minutes of treatment was optimal for horses to learn the lesson without becoming fatigued or restless. This involved the handler standing on one side of the horse, 50cm from the neck, facing the horse and positioned midway between the nose and shoulder, and applying tension to one rein, then releasing the tension when the horse turned its head to the side, laterally towards the handler, i.e., away from the pressure of the bit. Lateral movement, of more than 10cm, constituted a response and rein tension was released. If the horse did not move, lateral tension was gradually increased to the point that movement was induced. The horse was cued to give to pressure three times on one side and then the handler moved to the other side of the horse to repeat the exercise. Release of pressure was the only reinforcement used, the horses were not verbally praised or touched by the handler. The horse remained standing still during the treatment, with only lateral movement of the head and neck. Applying tension to one rein can signal the horse to move laterally. Horses that did move in the initial stages of treatment, stepped forwards. The average duration of each pre-conditioning treatment was 30 seconds per side.

Control horses (*n* = 37) were held for 8 minutes with the handler moving to the left and right side every minute, mimicking the movement of the handler in the treatment.

#### Trial phase

Immediately upon completion of the treatment and control phase, each horse was given to a different, trial handler (blinded to treatment allocation) and then led through the test corridor (5 m x 1.5 m) until the front feet were over the demarcated start line. The test corridor was 5 metres in length, marked with bollards at either end and designated by a fence and poles on the ground (See Fig 1) to guide movement in a straight line. The fence was used to prevent horses moving their hindquarters to the right when only one rein, in the first trial the left rein, was first picked up. The handler applied tension on the left rein in a caudal direction, cueing the horse to step backwards. When the horse stepped back, with any foot, all pressure was released. If the horse did not step backwards, rein tension was steadily increased until a step was taken. This procedure was repeated until the horse stepped out of the five-metre test corridor with the front feet.

The horse was then led, using only the lead rope attached to the head collar, around to the other end of the test corridor, up through the corridor and the handler halted the horse with the front feet beyond the start line, outside the corridor. The horse was led forwards and halted using the lead rope attached to the head collar and rein tension was not applied until the horse was halted and ready to begin the trial. The handler then moved to the right side of the horse and applied slight caudal tension on the right rein only, increasing tension steadily, if necessary, until the horse took a step backwards. As soon as the horse stepped back, the rein tension was released and the process repeated until the horse had exited the corridor with the front feet. Trials alternated between the left and right rein for a total of eight trials, beginning on the left, with four trials performed on each rein.

#### Recovery phase

The horse was held via a loose lead rope connected to the head collar for 5 minutes while recovery heart rate, heart rate variability and behavior were monitored.

### Statistical analysis

A split plot experimental design was used in which horses from both treatment and control groups received eight trials. Heart rate, rein tension, time and steps were compared using a mixed model General Mixed Model with trial (8 trials in total), treatment (treated or control) and side (left or right) as factors and horse identity as a random effect (REML command, GenstatTM, 17^th^ edition, VSN International Ltd, Waterhouse Street, Hemel Hempstead, UK). Prior to undertaking the REML analysis, the distribution of the data was visually inspected and tested for normality using a Shapiro-Wilk test. As all tests yielded a P>0.1, it was deemed that the data met assumptions for parametric analysis. Only the treatment.trial interaction was included in the final models. A visual inspection of the Polar heart rate data revealed some Type 1 errors [single transient spikes or troughs with great deviation from the surrounding data [27]] and corrections were made using the ‘low’ filter within the Polar software. Data were missing for the recovery phase of two horses.

The behavioral observation analysis used a generalised linear mixed model with binomial distribution for the number of events. A generalised model was used because behavioural elements were either seen/ not seen at each sampling point. Horse was used as a random effect and the probability of the behavioral event, as a proportion of the total number of observations, was reported.

## Results

### Behavioral observation

**Fig 2:**
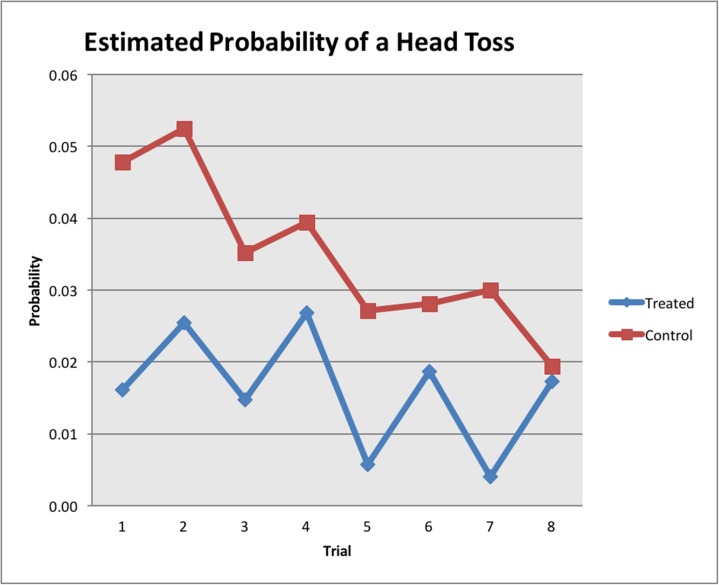
Estimated probability of a horse tossing the head during the trial phase of the trials. Trials 1, 3, 5 and 7 –left rein, trials 2, 4, 6 and 8 –right rein. Treated horses *n* = 23, control horses *n* = 33.

As the trials progressed, horses tossed their heads significantly less frequently (*P* = 0.015). For both treatment and control groups, head tossing was significantly more frequent during the trial phase of the experiment than in any other phase (*P* < 0.001) and control horses tossed their heads significantly more (*P* = 0.018) than treated horses. Among the treated horses, 71 per cent tossed their heads compared with 89 per cent of control horses. The right hand trials elicited more head tossing than the left hand trials for both the control and treated horses (see Fig 2). Horses did not toss their heads when led forward using the head collar and lead rope. All head tossing observed occurred when tension was being applied to the reins.

The number of times the horses contacted the handler varied significantly (GLMM, F_3, 198.3_ = 42.56, *P* < 0.001) over the different phases of the experiment, with the horses contacting the handler more frequently in the resting and recovery phases than in the treatment phase. The frequency of this behavioral response was not significantly different between the treated and control allocated horses (GLMM, F_1, 59.4_ = 0.91, *P* = 0.343).

Only fourteen horses, across both the treatment and control groups displayed pawing behavior, making analysis of this trait meaningless. The same reservations held for yawning, where only 58 events occurred. Analysis of ‘chewing on the bit’ failed to converge, as only eighteen horses displayed the behavior.

### Heart rate analysis

**Fig 3:**
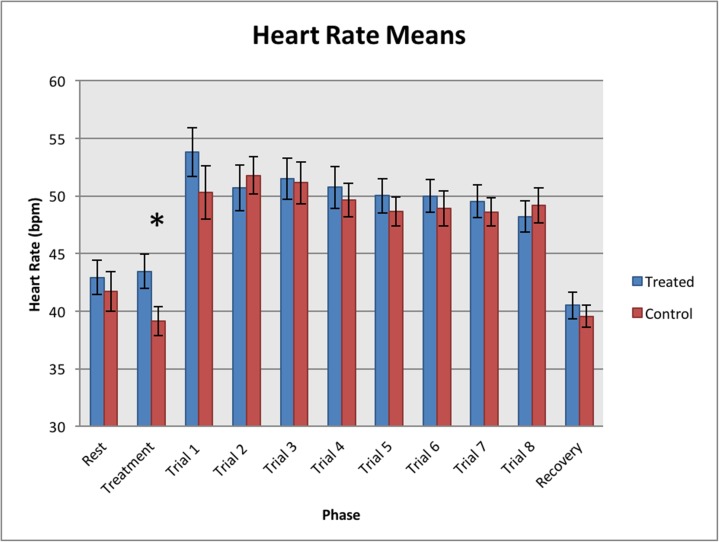
Heart rate means (bpm) for each of the experimental phases. Treated horses had significantly higher heart rates during the Treatment phase (*P* = 0.028).

Treated horses had significantly higher heart rates during the treatment phase than the control horses (GLM, F_1, 66_ = 5.01, *P* = 0.028). While a significant difference was found in heart rate across the trials (1–8) of individual horses (REML, F_6, 462_ = 4.07, *P* < 0.001), this difference was not significant between the left (Trials 1, 3, 5 and 7) and the right sides (Trials 2, 4, 6 and 8) (REML, F_1, 462_ = 0.15, *P* = 0.281) nor between the treated and control horses (RML, F_7, 462_ = 1.30, *P* = 0.246) (see Fig 3). During the recovery phase, both the treated and control group horses’ heart rates returned to their pre-trial resting rates (see Fig 3).

### Rein tension analysis

Both total, the sum of each rein tension measurement taken per second during the trial, and mean rein tension per trial were analysed because the latency to complete each trial varied greatly. For comparative purposes in the current study, a total figure was considered more useful than the mean as it more faithfully represented the tension applied by the handler.

**Fig 4:**
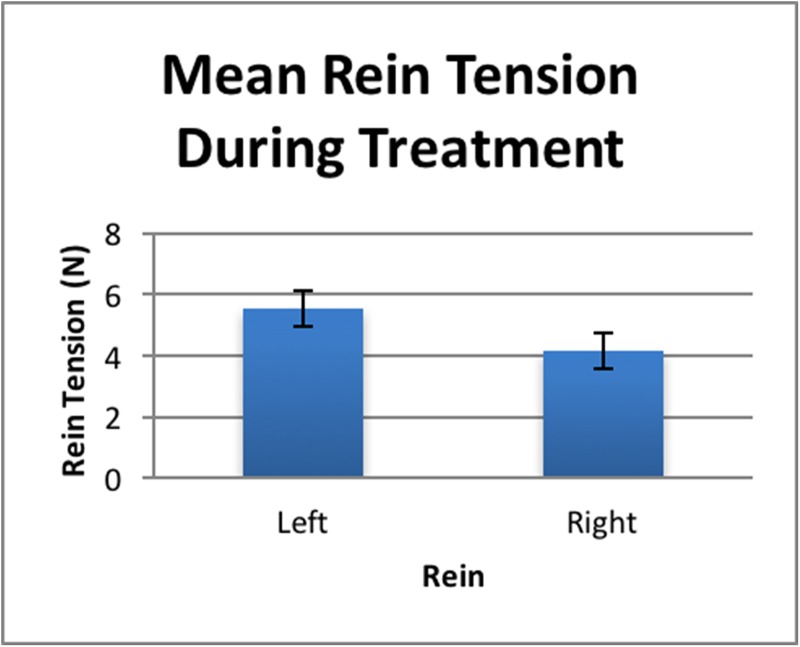
The significantly different (*t*-test, *P* = 0.009) mean rein tensions for left and right reins required to elicit a give-to-the-bit response during treatment (N = 31).

A significant difference between the mean rein tension required on the left and right reins was found during the treatment phase (*t*-test: *t*_30_ = 2.775, *P* = 0.009). Significantly more tension was required to elicit responses on the left rein (see Fig 4).

**Fig 5:**
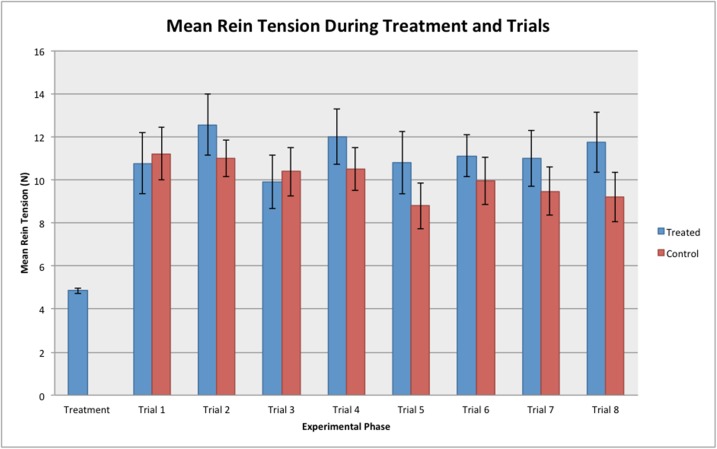
Mean rein tension (Newtons) during the treatment (n = 31) and trial (n = 68) phases of the experiment. No significant differences were found between the trials (REML, *P* = 0.481) or between the treated and control groups (REML, *P* = 0.349).

No significant difference in mean rein tension was found over the trials for each individual horse (REML, F_6, 462_ = 0.92, *P* = 0.481) (see Fig 5).

**Fig 6:**
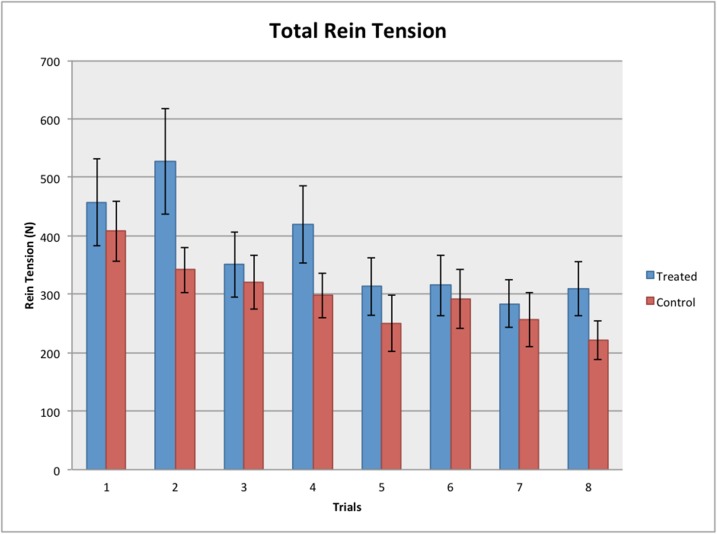
Total rein tension (in Newtons) required back-up the horse 5 metres (n = 68). Significant differences found between the trials (REML, *P* < 0.001) but not between the treated and control groups (REML, *P* = 0.146) or between the left and right reins (REML, *P* = 0.716).

A significant difference was found in the total rein tension exerted by the handler across the trials (REML, F_6, 462_ = 6.10, *P* < 0.001). As the trials progressed, horses required less rein tension to back the 5 metres through the test corridor (see Fig 6).

**Fig 7:**
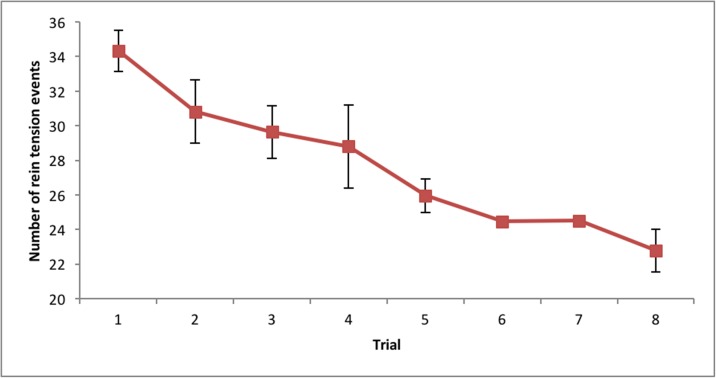
The decreasing (*P* < 0.001) mean number of rein tension events recorded for 68 horses during the standardised learning task (Trial 1–8) with standard error bars.

For both treated and control horses, there was a significant decrease in the number of rein tension events across all trials (*P* < 0.001), a slowly declining trend was detected from the following figures (Trial 1 = 43, Trial 2 = 31, Trial 3 = 30, Trial 4 = 29, Trial 5 = 26, Trial 6 = 24, Trial 7 = 25 and Trial 8 = 23 rein tension events) (see Fig 7). There was no significant difference in the mean number of rein tension events between the treatment and control groups (*P* = 0.294) during the trials.

### Time and steps

**Fig 8:**
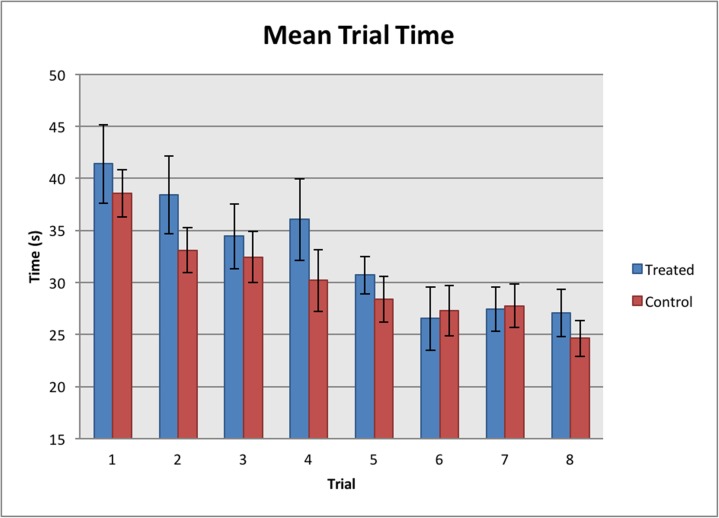
Mean trial time. Significant differences were found between the trials (REML, *P* < 0.001) but not between the treated and control groups (REML, *P* = 0.380).

Time for each trial was measured from the first step taken by the horse at the start of the corridor until the horse finally exited the corridor. The number of steps the horse took with the front feet were counted while the horse reversed along the 5m corridor. Throughout the series of eight trials, all horses travelled through the test corridor faster in subsequent trials (REML, F_1, 41.7_ = 41.67, *P* < 0.001; Fig 8). However, no significant difference in time taken was found between the treated and control groups (REML, F_1, 41.7_ = 0.77, *P* = 0.380; Fig 8).

**Fig 9:**
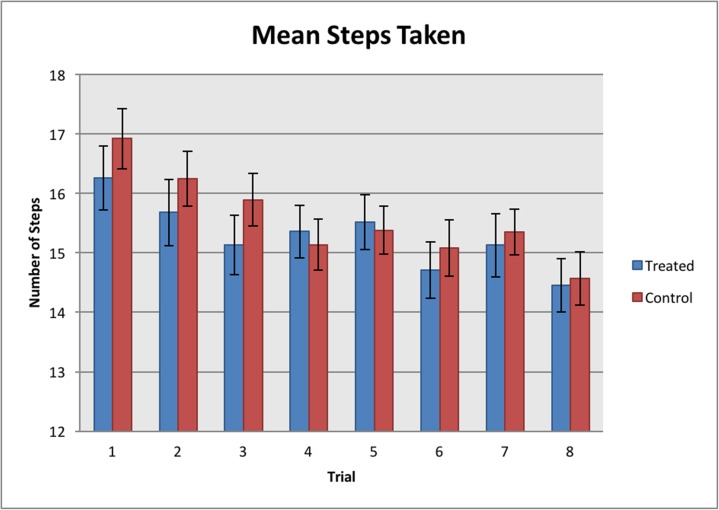
Mean number of steps taken per trial. Significant differences were found between the trials (REML, *P* < 0.001) but not between the treated and control groups (REML, *P* = 0.501). Significant differences were also found between the left (Trials 1, 3, 5 and 7) and right (Trials 2, 4, 6 and8) sides (REML, *P* < 0.001).

The number of steps taken by each horse decreased as the trials progressed (REML, F_6, 462_ = 10.65, *P* < 0.001; Fig 9), but there were no significant differences between the treatment and control allocated horses (REML, F_7, 462_ = 6.35, *P* = 0.501; Fig 9). There was a significant difference between the left and right sides (F_1, 462_ = 0.27, *P* < 0.001), with horses taking fewer steps when being handled on the right than the left side (see Fig 9).

## Discussion

To facilitate the production of safe riding horses, training that is consistent and adheres to learning theory is required [28]. Ideally, in any given lesson, the horse should learn the lesson in the fastest possible time with the least amount of associated stress, to produce a calm response. Developing easily accessible tools to assess the emotional state and arousal level [29] of the horse should optimize training, improve welfare and reduce wastage. An increase in heart rate can indicate increased arousal [30] as can behavioral parameters such as head tossing that, arguably, are easier to observe. This, together with appropriate reinforcement schedules (quantified by the use of rein tension meters to monitor the use of bit pressure and, more importantly, the release of such pressure) will help to define best practice in horse training.

In the current study, as the trials of the standardised learning task progressed, horses optimised their responses in several ways. They took fewer steps to complete the 5 m transit for each trial, indicating that they were increasing their stride length in each trial. They also completed the trials more quickly and with less rein pressure. Head tossing also reduced in a stepwise manner as the trials progressed, suggesting a reduction in stress. However, this was not reflected in the cardiac responses until the recovery phase. Head tossing occurred when rein tension was applied during the trials and the treated horses tossed their heads significantly less than the control horses. Pre-conditioning the horses to respond to rein tension resulted in fewer head tossing incidents during the trials phase of the lesson. Head tossing is seen as a conflict behavior that reduces perceived rideability and positive temperament traits in horses [31].

Effective use of negative reinforcement depends on timing the release of the applied pressure, and the difference between a *good* and an *average* trainer manifests chiefly in that ability. As McLean [32] argues, accurate timing of reinforcements should not only improve both training efficiency and performance by reducing the occurrence of conflict behaviors but also reduce horse wastage and improve welfare. The current findings, showing a significant reduction in the number of times horses tossed their heads as the trials progressed, indicate that horses learnt from the pre-conditioning exercise and that timing of the release was effective.

This study further supports the findings of others that bit pressure is aversive to horses. Christensen, Zharkikh, Antoine and Malmkvist [18] showed that horses voluntarily tolerated tensions of up to 11 N but did not habituate to greater tensions. In the current study, the required rein tensions steadily declined over the course of the eight trials suggests that, instead of habituating to bit pressure, the horses learned to avoid it. König Von Borstel and Glißman [33] and Heleski et al. [17] reported mean rein tension ranges between 9.1 ± 1.6 N and 21.7 ± 1.3 N and 3 to 20 N, respectively. These values correspond to the tensions recorded in the current study for the treatment and trials. The values in the present study were lower than those published by Clayton, Singleton, Lanovaz and Cloud [22] and Preuschoft, Witte, Recknagel, Bar, Lesch and Wuthrich [23] who reported ranges of 40 to 75 N. As the learning trials progressed, the mean required rein tension decreased. Similarly, the number of rein tension events decreased across trials, demonstrating that horses responded swiftly to the onset of pressure. This indicates that regardless of treatment, the horses did not habituate to pressure, but rather responded sooner to lighter pressure applied by the handler. This confirms that the handler’s pressure-release was providing negative reinforcement for the required operant response.

A few studies have examined rein tension at different gaits in ridden horses [[34]; [35]; [36]; [37]], while another measured tensions applied to a mechanical horse [38]. Only one study has investigated tensions required for learning a new lesson [37]. This showed that higher rein tensions were required for horses being long-reined or driven from the ground (using long lines connected to the bit) through a set pattern, than those being ridden through the same pattern. However, the authors pointed out that the long-reined horses may not have been familiar with this exercise and proposed that this may have accounted for the higher tensions required. While it is probable that long-reining itself does require more tension than riding, given the weight and length of the rein, resulting in a delayed and partial release of tension at best, it would be illuminating to repeat that study over an extend period to see if required rein tension reduced as the lesson was learned.

In a recent study of negative reinforcement by Ahrendt, Labouriau, Malmkvist, Nicol and Christensen [39] horses showed a significant decrease in the pressure required to complete each trial; in this case, the operant task was to step laterally from pressure applied to the hindquarter. Interestingly, these researchers used horses that were completely naïve to the exercise but still found significant laterality in the results, with more pressure being required on the right side of the horse. It was thought that this could be due to the position of the experimenter, who used the right hand to apply the pressure to both sides of the horse. The horses in the current study required more pressure on the left rein than the right during treatment which may be the result of habituation to bit pressure prior to the study or an inherent bias.

For the current study, it was decided not to randomize the order of the left and right trials, although it may useful to do so in future work. While the horses were all well-handled, horses are conventionally handled more on the left from the ground than the right [40]. The decision not to randomize the trial order was taken after carrying out a pilot trial with a small number of horses in which rein tension and heart rate parameters were monitored. This showed that horses that began trials on the right side required considerably higher rein tensions and had higher heart rates than their counter-parts beginning on the left rein. Ahrendt et al. [39] did randomize sides in their study but nevertheless found more pressure was required on the right side of the horse to obtain the desired response and concluded that learning was not transferred from one side to the other.

Interestingly, during treatment in the current study, horses required significantly less tension on the right to respond appropriately in the simple pressure-release task of give-to-the-bit. Our findings suggest that this may be the result of the horses trialling the newly learned give-to-the-bit behavior more on the right than the left. This could result in horses learning the initial give-to-the-bit lesson, on the right, with less pressure being required initially but then more pressure being requiring when being cued to move backwards from a stimulus with caudal, rather than lateral, rein tension [26]. As most experienced horses generally have had more handling on the left [40], the current cohort may reflect an acquired decrease in sensitivity to the left rein, as found during the treatment phase of the experiment.

The trialling of the newly learned behavior trained here may not occur to the same extent in a less formal training situation. Under non-experimental conditions, where the variables would not have to be isolated and measured, the handler could simplify the lesson by adding another form of reinforcement. This would possibly be positive reinforcement for each spontaneous step back or another form of negative reinforcement such as placing a hand on the horse’s chest, tapping lightly on the cannon bone with a dressage whip, moving in front of the horse or verbally cueing the horse to move back. Future studies could use an alternative follow-up lesson, such as the horse learning to load on to a trailer [41], to laterally yield the hindquarters using an algometer [39] or to walk over a bridge or tarpaulin. By not relying on bit pressure, these exercises would be significantly different from the treatment task. It would also be interesting to introduce other possible means of engaging the horse with alternative pressure-release exercises, such as head lowering from a halter [42] or a hindquarter yielding exercise.

Rietmann et al. [14] used a ground-work exercise (involving backing-up) that was similar to the current study, but considerably more challenging as the horses had to back-up for a full three minutes and various forms of reinforcement were used. Unfortunately, mixing positive, secondary positive and negative reinforcement makes the reinforcement schedule impossible to quantify and no attempt was made to do so. Rietmann et al. [14] reported that resting mean heart rates increased by 166 per cent with the initial backwards-walking exercise and that these rates came down to 85 per cent after some backing training was completed. However, such results are in stark contrast to the results of the current study, where mean heart rate rose by only 16 percent between the baseline collection period and the backing-up exercise. Rietmann et al. [14] suggest that the decrease they observed after training was the result of the horses now recognizing the predictability of the environment and learning that the stressor, the pressure from the handler, was predictable and controllable. Their horses walked backwards for a full three minutes, whereas those in the current study had to back-up for only 5 metres before walking forwards and around through the corridor again, to start the next trial. This more timely reward in the release of pressure may have improved learning outcomes, as the decrease in parameters including time, rein tension required, head tossing and steps taken indicate. The decrease in head tossing incidents suggests that horses are less stressed when given a pre-conditioning exercise in preparation for the lesson. Future research to accurately define the point at which the horse is optimally aroused for a given task should be considered a priority [43]. This optimal arousal state could be designated *the engagement zone*.

The collection of benchmark rein tension data is an important step towards improving horse welfare through trainer, coach and rider education. A quantitative definition of appropriate or ideal ‘contact’ and an understanding of how to correctly apply negative reinforcement so that horses become more responsive to pressure over time instead of desensitized to it will improve horse welfare. Whilst trainers, coaches and riders can all benefit from being made more aware of tension and also be incentivised to reduce overall rein tension over time [44], specifically targeting coaches and trainers has the potential to disseminate information widely and rapidly given their extension capacity.

## Conclusions

A simple pre-conditioning pressure-release exercise was used to engage the horses in an operant locomotory task. The exercise significantly increased heart rate, indicative of a moderate increase in arousal. Both the treated and control horses had similarly raised heart rates during the trials and both returned to baseline rates immediately following the trials. Both groups learned to avoid bit pressure by stepping back with longer strides and moving more quickly across the course of the trials. The treated horses exhibited significantly less head tossing than their untreated counterparts during the trial phase of the experiment. This suggests that engaging the horse prior to training may lay the foundation for a better learning experience.
